# The metabolic costs of meiotic drive

**DOI:** 10.1098/rspb.2025.0779

**Published:** 2025-07-02

**Authors:** Sasha L. Bradshaw, Enrique Rodríguez, Hanting Wang, Cyn Thea Yu, Cyril De Villiers De La Noue, Aadil Hafezjee, Andrew Pomiankowski, M. Florencia Camus

**Affiliations:** ^1^ Genetics, Evolution, and Environment, University College London, London, UK

**Keywords:** meiotic drive, mitochondria, respiration, metabolism, chromosome inversion, selfish genetic element, sexual selection, stalk-eyed fly

## Abstract

Selfish genetic elements, such as meiotic drive genes, disrupt Mendel’s law of equal segregation by biasing their own transmission, often at a detriment to the rest of the genome. Metabolic costs of the X-linked sex ratio (SR) meiotic drive were investigated in stalk-eyed flies (*Teleopsis dalmanni*). The experiments demonstrate that individuals with SR have reduced capacity for ATP synthesis. The disruption in mitochondrial function leads to compensation exhibited in increased basal metabolic rate and greater food consumption across a range of diets. The range of metabolic costs of drive was evident in males and females at a similar magnitude. The likely cause lies in the accumulation of deleterious mutations within the series of large inversions on the drive X chromosome, subject to low recombination and weak natural selection. In females, the drive chromosome had a dominant effect, with a single copy causing substantial metabolic compromise. There was little evidence of male-specific metabolic costs, nor evidence of greater effects of drive chromosomes on female metabolism. This suggests that direct metabolic costs from meiotic drive on spermatogenesis and from sexually antagonistic selection are relatively weak. Our results underscore the broad physiological impacts that selfish genetic elements have on host metabolism and fitness.

## 1. Introduction

Meiotic drivers are one of the most well-studied classes of selfish genetic elements that break Mendel’s law of equal segregation [1]. They have been identified on the autosomes and the sex chromosomes—the latter being more easily documented owing to the resulting bias in the progeny sex ratio [2–4]. Meiotic drive occurs via the preferential segregation towards the egg nucleus away from the polar bodies in females or through disabling non-carrier gametes post-meiotically in males, in both cases facilitating a transmission advantage to the drive allele [5]. The dysfunction or loss of sperm in male meiotic drive typically results in a direct cost to fertility [4,6,7]. This fitness reduction is particularly strong under conditions of sperm competition, both in defensive and offensive roles [6,8].

A range of indirect organismal costs are associated with male meiotic drive [1]. Loss of viability has been demonstrated in a range of species, both in females homozygous and heterozygous for drive alleles, as well as in drive-carrying males [9–12]. These costs are thought to originate from the propensity of drivers to position within low-recombination chromosomal inversions or other genomic regions subject to reduced recombination [3], resulting in weak selection and the accumulation of a high deleterious mutation load [13,14]. In addition, the spread of X-linked alleles depends on sex-specific selection (in part due to hemizygosity, which uncovers recessive alleles in males) and their asymmetric sexual transmission through the generations [15–17]. This is predicted to lead to the enrichment of sexually antagonistic alleles on driving X chromosomes that benefit male over female fitness, with greater than additive loss to female fitness in homozygotes [18].

The magnitude and direction of direct and indirect costs of meiotic drive have rarely been subjected to explicit empirical investigation. A means to gauge these costs is to examine the effects on metabolism [19,20]. Metabolism and metabolic traits are fundamental to life-history trait evolution, capturing the rate at which organisms transform and expend finite energy into growth, maintenance and reproduction [21–23]. Furthermore, metabolism is a useful ‘intermediate phenotype’, positioned between the level of the genotype and traditionally measured morphological and behavioural traits that contribute to fitness [24,25]. Although the association of metabolic traits with fitness is inevitably complex, it can provide a clear indication of where physiological processes are compromised by genetic variants, and are therefore likely to reduce fitness [26–30]. An advantage to studying metabolic traits is that they can be assessed in an equivalent manner in both sexes, allowing the investigation of sexual differences, which is key to the hypotheses investigated here.

In this study, three metabolic-related traits were measured to evaluate how meiotic drive directly in males and indirectly through its genomic architecture (through associated chromosomal inversions) in both sexes leads to costs linked to downstream consequences for fitness. These are investigated in the Malaysian stalk-eyed fly, *Teleopsis dalmanni*, which carries an X-linked meiotic drive system [11,31–35]. This species is known for its extreme sexual dimorphism, in which males have greatly exaggerated eyespans that are subjected to strong female mate preferences [34,36]. Males that carry the meiotic drive X chromosome, known as *Sex-Ratio* or SR, cause the dysfunction of Y-bearing sperm and sire female-only broods [5,8,37–39]. SR meiotic drive appears to be broadly stable across natural populations at a frequency of around 20% [33,40]. It is also present in the sister species *Teleopsis whitei*, which suggests a common origin estimated at around 4 million years ago, though sequence evidence is lacking to support this hypothesis [40,41].

*T. dalmanni* drive males curiously do not suffer from reduced fertility despite the destruction of half of their gametes [42]. They pass on the same number of sperm per ejaculate as wild-type males and have equal fertility when in competition with wild-type males [43,44], although an earlier study found some now contested evidence for a fertility difference [35]. This can be explained by the adaptive enlargement of testes in drive males, which is evident in the primordia of the adult fly testes upon eclosion from the pupal stage, accelerated growth rates during early adult development, and increased size of testes in sexually mature flies [45]. This view is complicated by a recent experimental evolution study that reported reduced SR frequency under multiple mating, which points to SR males faring poorly when subject to high levels of mating competition [46]. A number of other traits associated with the meiotic drive X chromosome are thought to be detrimental to fitness. Drive males have reduced eyespan under laboratory conditions and in the field [11,31,34,42,47], smaller accessory glands [42]—though this is not well established [45]—and mate less frequently than wild-type males [11]. Drive females do not have reduced eyespan [31,47] but have lower fecundity compared to wild-type females [48]. Both sexes have reduced egg-to-adult viability when they carry the drive chromosome [11]. These phenotypes and fitness measures highlight the breadth of meiotic drive-induced costs, emphasizing the value to investigate the underlying metabolic consequences of harbouring drive across both sexes.

Three diverse measures of metabolic function were assessed to examine the impact of meiotic drive on metabolic life history. Mitochondrial function was measured by monitoring oxygen consumption in thoracic tissue using an O2k-Fluorespirometer [49,50]. Whole-organism resting metabolic rate was assayed through CO_2_ production measurements using a MAVEn-FT (Multiple Animal Versatile Energetics Flow-Through System, Sable Systems International, Las Vegas, NV). Finally, nutrient acquisition under resting conditions was assessed through the consumption of different diets using a capillary feeder (CAFE) assay [51]. The primary aim was to compare flies carrying drive and wild-type chromosomes, as well as between heterozygous and homozygous females, to test the prediction of general deleterious effects associated with the drive X chromosome. The hypothesis that dysfunction caused by meiotic drive during spermatogenesis leads to direct metabolic consequences was tested by comparing the sexes, with the expectation that males would exhibit more pronounced effects. The hypothesis that there is an accumulation of sexually antagonistic effects linked to drive was tested in the same way but with the reverse expectation that female metabolism would be more strongly impacted.

## 2. Material and methods

### (a) Experimental fly generation

The wild-type, standard (ST) stock was collected in 2005 from the Ulu Gombak Valley, Peninsular Malaysia [31,52]. Flies with the X^SR^ genotype were collected in 2012 from the same location [31]. Since 2021, they have been maintained by crossing homozygous SR females (X^SR^/X^SR^) to wild-type males (X^ST^/Y) to produce drive males (X^SR^/Y), who are then mated back to X^SR^/X^SR^ females to generate the next generation of homozygous SR stock females. Note that the breeding procedure for ST and SR stock means that the autosomal and Y genetic content is regularly mixed and does not differ between flies used in these experiments.

Experimental ST males (X^ST^/Y) and homozygous ST females (X^ST^/X^ST^) were collected on egglays (petri dishes with a damp cotton pad and pureed sweetcorn as food) from cages housing X^ST^/X^ST^ females and X^ST^/Y males. The egglays were incubated at 25°C, and the emerging flies were collected. The same procedure was followed to collect SR males (X^SR^/Y) and heterozygous females (X^SR^/X^ST^) from cages housing X^SR^/X^SR^ females and X^ST^/Y males and to collect homozygous SR females (X^SR^/X^SR^) from cages housing X^SR^/X^SR^ females and X^SR^/Y males. We used standardized procedures in our collection and testing of individuals for the experiments, but inevitably there is variation as the different genotypes were generated in separate crosses. A range of corrections were applied in the statistical analyses to control for such variation (see below).

The number of adult flies in the parental cages was equivalent in the three crosses to ensure that approximately similar densities of eggs were laid, limiting variation in larval density. There were inevitable differences between the ‘social environment’ in the production of offspring as genotypes differed, and in particular, in the production of SR homozygotes, as drive male parents only produce female offspring. However, we have no evidence that the sex ratio among competing larvae influences adult phenotype (unpublished data). Age was measured from eclosion, with an inaccuracy of 1−3 days as flies were not collected every day. Adult flies were collected and held in single-sex containers. All experimental flies were virgins and sexually mature when used in experiments [53,54].

### (b) Measuring mitochondrial function through high-resolution respirometry

Flies used in the experiment were aged approximately 50−60 days post-eclosion. The preparation of *T. dalmanni* thoracic tissue for mitochondrial function analysis was adapted from published methods [50,55]. Whole thoraces were dissected in 1.5 ml ice-cold MiR05 respirometry buffer (0.5 mM EGTA, 3 mM MgCl2.6H2O, 60 mM lactobionic Acid, 20 mM taurine, 10 mM KH2PO4, 20 mM HEPES, 110 mM D-sucrose and 1 g l^−1^ BSA, pH 7.1). The muscle fibres were lightly homogenized in 150 μl of MiR05, of which 50 μl were added in a calibrated O2k-Fluorespirometer (Oroboros Instruments, Innsbruck, Austria) with 2 ml MiR05. The remaining homogenate was frozen and kept for subsequent protein content analysis using a QuantiPro BCA Assay Kit (Sigma-Aldrich).

Pyruvate (10 mM) and malate (2 mM) (Complex I substrates) were then added to the Oroboros sample chamber, and the LEAK state was recorded after 15−20 minutes. ADP (5 mM) was added to reach the OXPHOS state. Cytochrome c (10 mM) was added to assess mitochondrial membrane integrity, and samples with >20% increased O_2_ consumption were discarded. Glutamate (10 mM), succinate (10 mM) and glycerophosphate (10 mM) were added sequentially, recording O_2_ fluxes at each state. Maximum uncoupled respiration was determined using 0.5 μM FFCP. Respiration was inhibited by adding rotenone (0.5 μM), followed by malonic acid (5 mM), and antimycin A (2.5 μM), estimating the residual oxygen consumption (ROX). ROX was set as the baseline (O_2_ consumption = 0), and other states were compared to ROX-corrected states. Finally, Complex IV activity was measured using ascorbate (2 mM) and TMPD (0.5 mM), followed by inhibition with sodium azide (100 mM).

Following Oroboros runs, both respiratory control ratio (RCR) and Complex I-linked efficiency were calculated. The RCR was calculated by dividing the oxygen consumption when ADP was added by the LEAK state (State 3/State 4). RCR is a complex function that reflects changes in many aspects of oxidative phosphorylation. High RCR values indicate an elevated capacity for substrate oxidation, low proton leak and high respiratory capacity available to phosphorylate ADP to ATP [49,56]. The activity of respiratory Complex I was calculated as the percentage decrease upon the addition of the inhibitor rotenone in the uncoupled state. Complex I is a crucial component of the electron transport chain, pumping protons into the inter-membrane space that largely re-enter through the ATP synthase [49].

Each Oroboros instrument is equipped with two chambers, and two instruments were available for this experiment, enabling the simultaneous testing of four genotypes. Due to this logistical limitation, only wild-type and drive males, as well as homozygous wild-type and homozygous drive females, were included in this study. Heterozygous females were not tested in this experiment.

### (c) Measuring whole-organism metabolic rate

Whole fly resting metabolic rate was measured using a MAVEn-FT (Multiple Animal Versatile Energetics Flow-Through System; Sable Systems International, USA). Individual flies (41–72 days post-eclosion) were weighed and then placed into metabolic chambers coupled to an external CO₂ analyser (LICOR 850; LI-COR, USA). Fly activity was measured via the presence of three infrared beams below each chamber, recording movement every time the beams were broken. The chambers (length: 3 cm, diameter: 1.3 cm) were large enough so that flies could move around but small enough that they could not undertake behaviours such as flying or foraging. The CO_2_ concentration in each chamber was measured during airflow for 120 seconds at a flow rate of 30 ml min^−1^. The system monitored 16 individual chambers consecutively, with three or four measurements for each chamber, over a 3-hour cycle.

The multiplexed configuration included 15 chambers containing flies and one chamber left empty as a control to confirm that there was no interference from extraneous variables. Eight separate trials were conducted, each involving a different set of 15 individuals. Three individuals of the five genotypes were tested in each trial: two male genotypes, X^ST^Y and X^SR^Y, and three female genotypes, X^ST^X^ST^, X^SR^X^ST^ and X^SR^X^SR^. At the beginning of a trial, all individuals were lightly anaesthetized with 5 ppm of CO₂, weighed (mg) and then allowed to wake and acclimate for 30 minutes before being placed inside the MAVEn-FT system.

### (d) Measuring dietary consumption

Food consumption was measured using a CAFE assay [51] adapted for use in stalk-eyed flies. Experimental individuals (20–25 days post-eclosion) were placed individually in vials (20 ml) containing a base layer (6 ml) of 0.8% agar to provide a source of hydration with no caloric content. The top of the tube was secured with a sponge bung penetrated by a pipette tip cut to fit a capillary tube. Flies remained in their agar vials overnight. The following morning, a 20 μl glass capillary filled with the allocated dietary treatment was inserted into each individual vial (electronic supplementary material, figure S1). Dietary treatments used in this experiment were a mix of microbiology yeast extract and sugar, but all with a final concentration of 32.5 g l^−1^. The three diet treatments used were high protein (1 : 1 yeast : sugar), low protein (1 : 2 yeast : sugar) and sugar alone (10% solution). Blank controls (empty vials containing a liquid capillary but with no fly) were used as controls to estimate the volume of liquid lost through evaporation alone.

All vials were placed in an incubator (PHCI-MLR-352H Climate Chamber) at 25°C and 90% humidity to maintain moisture in the vials and minimize capillary liquid evaporation. The liquid meniscus at the top of the capillaries was marked, and the decline over known time periods due to consumption and evaporation was recorded. The physical distance between these marks was measured using digital callipers to obtain the volume of liquid consumed (μl) from the 20 μl capillary. Consumption was measured, and food was replaced after 20, 40 and 60 h, resulting in the total consumption (calculated as the sum across the 3 days).

The five genotypes were tested, two male and three female, as listed in measuring whole-organism metabolic rate section above. The thorax of ice-anaesthetized flies was measured upon completion of the experiment using an Infinity Capture video microscope attached to a computer equipped with NIH image software (FIJI - IMAGEJ, v. 2.1.0/1.53 c). The thorax was measured from the prothorax anterior tip along the midline to the joint in between the thorax and metathoracic legs [57].

### (e) Statistical analyses

All statistical analyses were carried out using R Studio (v. 2023.12.0). We used linear models using either the ‘*lm*’ or ‘*lmer*’ function in R, depending on whether the models had random effects. Statistical models are reported in full in the electronic supplementary material (SI Models).

#### (i) Mitochondrial function

Oxygen consumption data were normalized by protein content within the Oroboros software (DatLab v7.8). Sex and genotype were accounted for in all models as fixed effects. Interactions between variables were tested, and the model was reduced by removing interaction terms that were not significant. The response variables RCR and Complex I function are reported in the text, while others are reported in the electronic supplementary material (SI Models, §5).

#### (ii) Whole-organism metabolic rate

MAVEn data files were processed using Sable Systems software (ExpeData v.1.7), which extracted the 30 second window (within the 2-minute reading) with the lowest CO_2_ production and corresponding activity values of individual flies. Weight, sex, activity level, age of fly and genotype were accounted for in all linear regression models. Interactions between variables were tested, and the model was reduced by removing interaction terms that were not significant. Three or four measurements were made from the same individual. To account for this, individual sample ID was added as a random effect. Variations between flies in weight, activity and age were added as covariates in all analyses. Weight and activity both had positive effects on CO_2_ production, whereas age did not. Marginal means are reported in the text where differences were observed.

SR is on the X chromosome, and this results in five genotypes; three for females (X^ST^X^ST^, X^SR^X^ST^ and X^SR^X^SR^) and two for males (X^ST^Y and X^SR^Y). Given the differences in genotype numbers between the sexes, a two-step analysis was used to properly assess the effect of all genotypes on a given trait. In the first, the five genotypes were reduced to two genotype categories, ST or SR, for each sex. Heterozygous females and SR homozygous females were pooled in the female SR category (i.e. carrying a drive chromosome). Here, we examined the effect of genotype (SR/ST) and sex, plus their interactive effect on metabolic rate. The second analysis was aimed to dissect dominance among the female genotypes, which compared the effect of the three female genotypes on metabolic rate. A further analysis is given in the electronic supplementary material, treating all five genotypes as separate experimental units (SI Models, §3d). The results justified the pooling of drive-carrying females.

#### (iii) Dietary consumption using the capillary feeder assay

Prior to the analysis of the data, the mean food loss due to evaporation (blank vials with no fly) was subtracted from the consumption of individual flies. The data were log-transformed to meet statistical model assumptions of normality. The full models included consumption values as a response variable, with thorax (as a proxy for body size), sex, treatment (diet type) and genotype as fixed factors. Interactions between variables were tested, and the model was reduced by removing interaction terms that were not significant. The random effect of ‘box’ was also included to control for any batch differences across experimental runs. Genotype was assessed using the two methods outlined in the whole-organism metabolic rate section.

## 3. Results

### (a) Mitochondrial function is genotype-dependent

**Figure 1.**
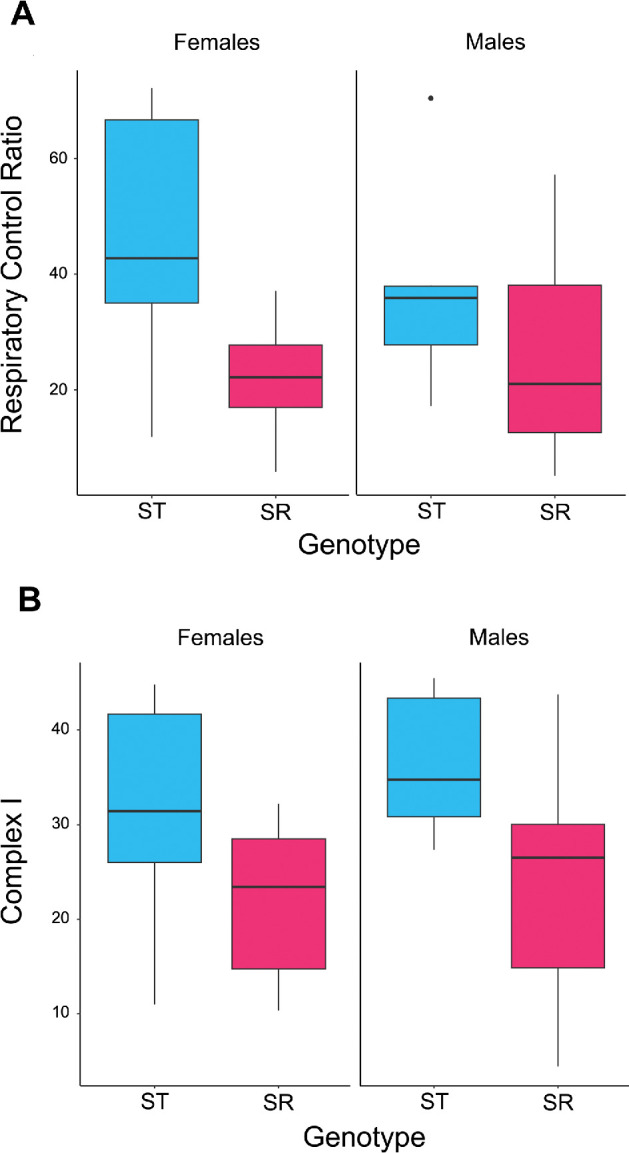
(A) Respiratory control ratio (RCR) and (B) percentage of Complex I contribution to respiration in females (left) and males (right) for ST (blue) and SR (pink) genotypes. Females with the SR genotype were SR homozygotes. The central line in each box represents the median, the box indicates the interquartile range and the whiskers represent the 95% CI.

Genotype affected mitochondrial activity as measured by the RCR (F_1,23_ = 6.394, *p* = 0.019), where ST individuals had higher RCR (42.540 ± 5.669) than SR individuals (24.375 ± 4.201; figure 1A). There was no difference across the sexes (F_1,23_ = 0.151, *p* = 0.702) and no interaction between genotype and sex (F_1, 22_ = 0.995, *p* = 0.329).

In addition, ST individuals displayed a higher Complex I contribution to respiration (33.902 ± 2.815) than SR individuals (22.762 ± 3.180; F_1,23_ = 6.741, *p* = 0.016; figure 1B). Again, there was no difference across the sexes (F_1,23_ = 0.512, *p* = 0.482) and no interaction between genotype and sex (F_1,22_ = 0.115, *p* = 0.738). All other respiratory states of the electron transport system did not vary between SR and ST individuals (*p* > 0.05, electronic supplementary material, SI Models §5).

### (b) Whole-organism metabolic rate is sex- and genotype-dependent

**Figure 2.**
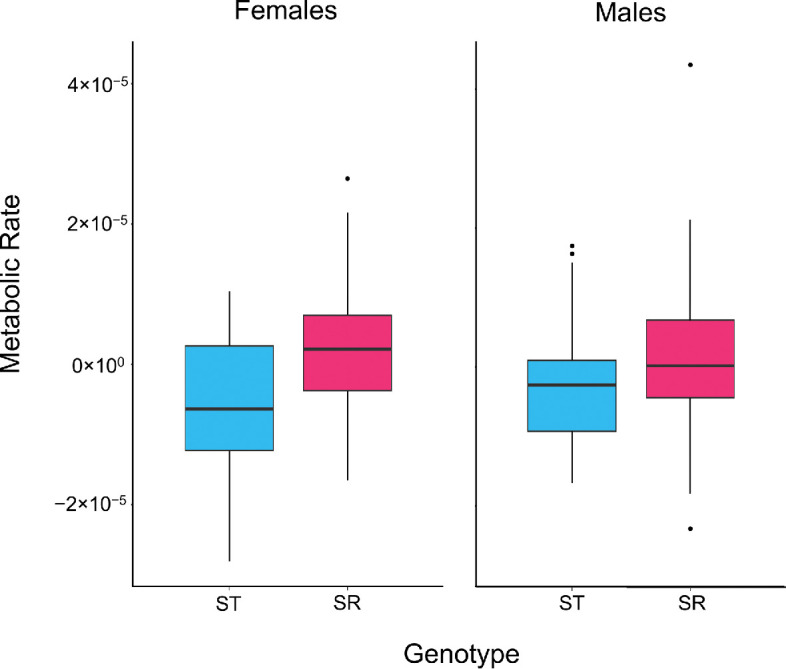
CO₂ (µl min^−1^) produced as a measure of metabolic rate in females (left) and males (right) for ST individuals (blue) and SR individuals (pink). Basal metabolic rate is plotted as residuals after accounting for body size, activity and age variation. Heterozygotes and SR homozygous females are pooled. The central line in each box represents the median, the box indicates the interquartile range and the whiskers represent the 95% CI.

In our first analysis, genotype was found to alter respiration (F_1,91.79_ = 12.424, *p* > 0.001), with a higher level of CO_2_ production in individuals carrying SR chromosomes (2.05 × 10^−4^ ± 1.16 × 10^−5^) compared to those with only ST chromosomes (1.60 × 10^−4^ ± 1.31 × 10^−5^; figure 2). Respiration also differed between the sexes (F_1,112.52_ = 8.669, *p* = 0.004), with males (1.96 × 10^−4^ ± 1.16 × 10^−5^) having greater respiration than females (1.69 × 10^−4^ ± 1.13 × 10^−5^; figure 2). There was no interaction between genotype and sex (F_1,113.59_ = 0.102, *p* = 0.750).

In the analysis above (as described in the methods), female SR homozygous and heterozygous genotypes were pooled as both genotypes carried drive. This was followed by a female-specific analysis, where it was found that respiration differed among the three genotypes (F_2,71.35_ = 6.897, *p* = 0.002; electronic supplementary material, figure S1). SR homozygous females showed the same level of respiration as heterozygotes (Tukey’s HSD: *t* = −0.070, *p* = 0.998, d.f. = 66.8). Both SR homozygotes (*t* = 3.337, *p* = 0.004, d.f. = 71.7) and heterozygotes (*t* = 3.199, *p* = 0.006, d.f. = 56.8) had elevated respiration compared to ST homozygotes. In addition, there was no difference in the cross-sex comparison of all individuals with drive chromosomes (SR males, female heterozygotes and female SR homozygotes; all *t* ≤ 0.264, *p* ≥ 0.1232).

### (c) Dietary consumption using the capillary feeder assay is diet- and genotype-dependent

**Figure 3.**
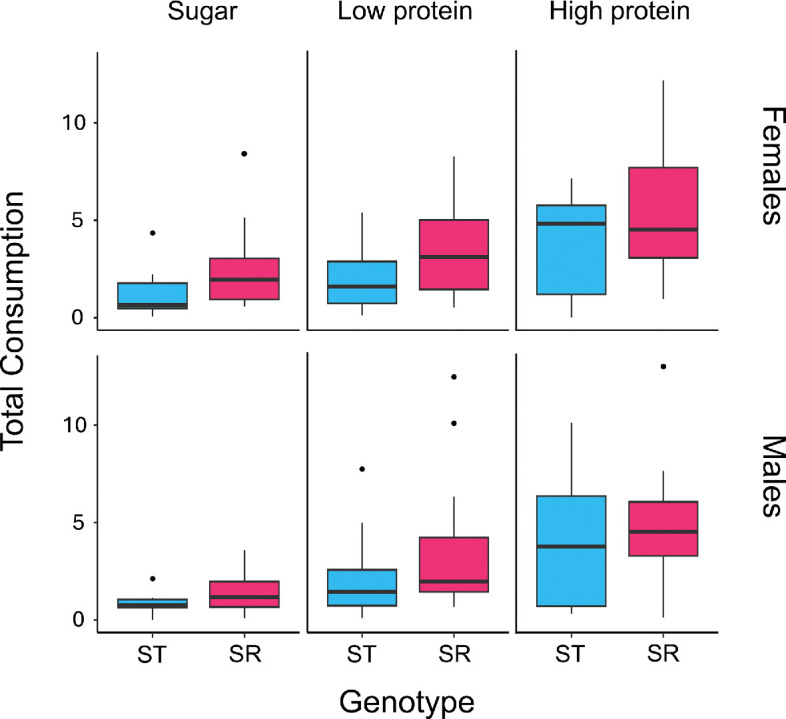
Total consumption (μl) of liquid diet over the 60-hour experiment (plotted as residuals after correcting for body size) of sugar (left), low protein (middle) and high protein (right) by females (top) and males (bottom). Genotypes are ST (blue) and SR (pink). Heterozygotes and SR homozygous females are pooled. The central line in each box represents the median, the box indicates the interquartile range and the whiskers represent the 95% CI.

Pooling SR heterozygotes and homozygotes, genotype-altered food consumption (F_1,224.96_ = 7.966, *p* = 0.005), as individuals carrying SR chromosomes (7.559 ± 0.513) consumed a greater total amount of food than individuals with only ST chromosomes (4.855 ± 0.520). There was no effect of sex on food consumption (F_1,223.68_ = 3.377, *p* = 0.067). Consumption varied with diet (F_2,224.01_ = 21.401, *p* < 0.001), with more of the high protein diet consumed than the low protein diet (*t* = 2.747, *p* = 0.018, d.f. = 225), which in turn was consumed more than the sugar diet (*t =* 3.784, *p* < 0.001, d.f. = 224; figure 3). There was no interaction between diet and genotype (F_2,222_ = 0.264, *p* = 0.768) or between genotype and sex (F_1,223.53_ = 0.033, *p* = 0.855).

Limiting the analysis to females (as in the whole-organism metabolic rate section), consumption differed among the three genotypes (F_2,137.22_ = 3.726, *p* = 0.027; electronic supplementary material, figure S2). *Post hoc* comparisons showed that heterozygotes consumed more than ST homozygotes (*t* = −2.648, *p* = 0.024, d.f. = 138), but other comparisons were not significant. Consumption again varied with diet (F_2,136.97_ = 10.900, *p* < 0.001). The high protein diet was consumed more than the sugar diet (*t* = 4.665, *p* < 0.001, d.f. = 137). There was no difference between the consumption of high protein and low protein diets (*t =* 2.359, *p =* 0.051, d.f. = 137) or low protein and sugar diets (*t =* 2.324, *p =* 0.056, d.f. = 137), though both comparisons were borderline significant. There, again, was no interaction between diet and genotype (F_4,132.99_ = 0.267, *p* = 0.899). In addition, there was no difference in consumption for the cross-sex comparisons (all *t* ≤ 1.643, *p* ≥ 0.231) of all individuals with drive chromosomes (SR males, female heterozygotes and female SR homozygotes).

## 4. Discussion

The X-linked SR meiotic drive system in stalk-eyed flies has distinct metabolic consequences. We provide evidence that drive flies suffer mitochondrial dysfunction, and this dysfunction is compensated via an increase in basal respiration rate. SR flies also consume more food per unit of time across a range of diets, indicating further compensatory mechanisms to counteract their less efficient metabolism and a potential inability to sufficiently utilize nutrients. These results provide the first demonstration of how metabolic function can be corrupted by meiotic drive. All the markers of stress within the metabolic system occurred in females as well as males carrying the drive chromosome, despite the direct disruption of meiosis in stalk-eyed flies being limited to spermatogenesis. This strongly points to the genomic inversions on the driving X chromosome being linked to a raised mutational load, which causes a range of metabolic costs.

Mitochondrial function was measured through high-resolution respirometry using an O2k-Fluorespirometer (Oroboros Instruments, Austria). This measures the flux through the electron transport chain during OXPHOS using a substrate-uncoupler-inhibitor titration protocol (see electronic supplementary material for details). Two aspects were found to be compromised. Individuals carrying meiotic drive had a lower RCR (figure 1A), indicating the uncoupling of oxidative phosphorylation from ATP synthesis [58]. They also presented reduced Complex I substrate oxidation contribution to respiration (figure 1B). Complex I is the first and largest unit in the respiratory chain and the primary contributor to the proton motive force that facilitates ATP synthesis [59]. Other mitochondrial states tested were not found to differ between SR and ST individuals (electronic supplementary material, SI Models §5). A possible cause of low RCR and Complex I dysfunction is mutations in the nuclear DNA encoding mitochondrial subunits or accessory proteins located within the driving X chromosome inversions. For example, several X-linked mutations associated with Complex I dysfunction have been characterized by various pathologies [60,61], including the NDUFA1 gene implicated in mitochondrial encephalomyopathy [62]. These possibilities could be tested in future bioinformatic work comparing SR and wild-type sequence data [63], specifically looking for evidence of disruption to coding sequences of nuclear genes involved in mitochondrial function or for differential expression of nuclear and mitochondrial genes involved.

There were no sex differences in the RCR or Complex I measurements, but the sample size was not large, which limits the assurance of this result. However, it is noteworthy to mention that the number of samples tested in this experiment is typical of protocols used in this field [64–68]. In addition, the flies used in this experiment were virgins, which suggests it would be interesting to investigate mitochondrial metabolism in mated flies. Previous studies have reported reduced sexual dimorphism in virgin metabolic rate [69]. A potential shortcoming is that the O2k assessment was limited to female homozygotes, meaning that the degree of dominance of drive effects on mitochondrial activity in females cannot be discerned. This could be investigated as part of future work. Another area to investigate is the male reproductive tissues, where the drive genes causing disruption of spermatogenesis are active (i.e. the testes and accessory glands) [70]. These tissues are expected to show elevated dysfunction compared to thorax musculature due to the direct effect of drive, which might be measurable through mitochondrial deficits in RCR, Complex I or other elements of the respiratory chain. Previous studies in the *t* haplotype meiotic drive system in mice provide valuable parallels. Mutant *t^n^* sperm show increased aerobic metabolism (exemplified by a reduced NADH/NAD ratio), which is thought to put them at a selective advantage because it increases motility, maturation and fertilization [71]. In some cases, drive not only causes dysfunction in wild-type sperm, but also causes drive sperm to experience pleiotropic collateral damage [6]. This does not appear to be the case in *T. dalmanni,* as SR males show no reduction in sperm competitiveness or paternity gain in competition with wild-type males [43,48], though experimental evolution suggests weaker fertility under high multiple mating [46]. The most plausible explanation is the compensatory enlargement of SR male testes, with a concomitant equal sperm count per ejaculate when compared to wild-type males [42,44]. How these adaptive changes alter mitochondrial function in these tissues remains to be investigated. In contrast, female reproductive tissues are not expected to be differentially affected compared with somatic tissues, as meiotic drive has no known downstream consequences for oogenesis in stalk-eyed flies. Mitochondrial function again remains to be assessed.

The whole-organism metabolic rate of stalk-eyed flies was examined using a MAVEn-FT system, which takes repeated measurements of CO_2_ production of resting flies over a 3-hour period. Respiration was higher in drive individuals. Female drive heterozygotes and homozygotes were tested alongside wild-type homozygous females, which demonstrated that the SR chromosome has a dominant effect on respiration, as females with a single SR chromosome produced as much CO_2_ as those with two (figure 2). Likewise, there was no difference between drive males and the two female genotypes carrying drive (figure 2). These findings suggest that less efficient mitochondrial function induces a compensatory elevated metabolic rate in drive-carrying individuals, both male and female. This is consistent with the notion that most of the metabolic costs arising are associated with the mutational load in the SR chromosome inversions, as was seen in direct measurements of mitochondrial activity. Increases in CO_2_ production of a similar dimension have been noted in other insects, between virgin and mated flies [69], associated with sexually selected weapons [72] and when there is an incompatibility between the nucleus and the mitochondria [73–76], and other species [74,76]. However, opposing results were found in mice, as females carrying the *t* locus were found to exhibit a lower resting metabolic rate compared to wild-type [77]. This association led the latter authors to argue that reduced metabolism is an adaptation in drive female mice to compensate for smaller litter sizes [4], as a lower metabolic rate has been linked to increased longevity [78], thereby extending the number of litters they sire during their lifespan [78]. The decrease in metabolic rate with body size is not observed in males carrying the *t* locus, so it is not a direct consequence of drive; rather, it is a female-only phenomenon, which aligns with the adaptation hypothesis [78]. We note that whole-organism respiration in stalk-eyed flies was greater in males than in females after taking account of differences in body size (with which metabolic rate scales). Sex differences in metabolic rates are common, reflecting the different life histories and management of energy budgets [69,72]. However, there was no variation in this difference and with carriage of the meiotic drive chromosome.

A final test of metabolic costs arising through meiotic drive measured food intake over a range of diets using the CAFE assay. Food consumption is a good proxy for metabolic-reliant phenotypes, given its involvement in fitness and lifespan across many species, including *D. melanogaster* [79,80], and its association with metabolic rate [81–83]. SR individuals consistently consumed more (figure 3). Consumption varied markedly with diet, and intake increased with greater amounts of protein in the diet provided. However, diet type had no impact on the difference between SR and wild-type flies. This suggests that metabolic deficiency among flies carrying drive chromosomes is compensated by greater food intake. Even though the value of different food sources varies with the reproductive demands of females and males [84,85], this was not observed in this assay. Typically, females prefer diets with greater protein content (for egg production), and, in contrast, males prefer carbohydrates to power male-male competition and sexual display. Even if this is the case in stalk-eyed flies, drive individuals did not show consumption modification in response to their mitochondrial dysfunction across different diets other than an overall increase in consumption. In addition, as in the whole-organism respiration tests, there was no difference between female heterozygotes and homozygotes, indicating dominance of the dysfunction caused by meiotic drive. Nor did these two female genotypes differ in consumption compared to drive males (figure 3). It was surprising that there was no difference between females and males across the three diets (figure 3). This may reflect the virgin status of the flies used. There was a similar lack of sexual dimorphism in virgin *Drosophila melanogaster* but a large sex difference among mated flies [84], which suggests a further study for dietary differences between mated drive and wild-type stalk-eyed flies of both sexes.

Altogether, these results point to the preponderance of indirect mutational load costs being linked to meiotic drive in a sex-independent manner. This does not rule out the existence of direct effects of meiotic drive in males that reduce metabolic function, nor the possibility of selection for linked sexually antagonistic alleles that depress female metabolic function [18]. What it suggests is that these effects are relatively less important and subsumed by the indirect mutational load held in the multiple inversions that cover almost all of the SR X chromosome in *T. dalmanni* and other examples of X-linked drive [3,40]. Metabolic function measured in this study concerned somatic tissue, whole-organism respiration and dietary consumption. It may be that reproductive tissues where meiotic drive takes place (i.e. male testes) or gametogenesis in general (i.e. including ovarian tissue) have different metabolic responses reflecting the direct effect of meiotic drive genes or sexually antagonistic gene expression, respectively. This possibility suggests future work.

## 5. Conclusions

This research provides the first evidence of the range of metabolic costs associated with a meiotic drive system in stalk-eyed flies. The costs uncovered include mitochondrial dysfunction (lowered RCR and reduced Complex I contributions to respiration), metabolic inefficiency (higher basal metabolic rate) and increased food consumption across a range of diets (varying in protein content). The latter two are likely to be compensatory mechanisms arising from mitochondrial dysfunction. The costs were of equal scale across the sexes even though the direct action of meiotic drive only manifests as a loss of gametes in male spermatogenesis. This closely mirrors measures of egg-to-adult viability which revealed almost identical selective coefficients in males and females possessing drive or wild-type X chromosomes [11]. This supports the hypothesis of predominant indirect viability costs associated with meiotic drive. These are likely to arise from genes linked to meiotic drive on the X chromosome. The driving X chromosome contains a number of large genomic inversions, which restrict its recombination with the wild-type X chromosome. As the meiotic drive X chromosome is at a lower frequency than the wild-type in natural populations (approx. 20%), it is subjected to weak selection and the accumulation of deleterious mutations which impact metabolic function. By comparing heterozygous and homozygous females with hemizygous males, the analysis found no evidence of predominant recessive effects in females as predicted by sexually antagonistic selection. This also mirrors the study of egg-to-adult survival which reported additive (i.e. *h* approx. 0.5) rather than recessive effects in females [11]. Why this contrasts with other studies reporting recessive effects of meiotic drive in females is not clear [3,12]. Surprisingly, there was little evidence of greater metabolic deficits in males, even though they are subject to strong sexual selection and have exaggerated secondary sexual traits [52,86,87] nor was there a greater effect of drive in males. A further analysis of reproductive tissues is needed to evaluate whether male-specific metabolic costs are evident where the meiotic drive genes themselves are active. Taken together, these results offer new insights into the metabolic and energetic underpinnings of harbouring meiotic drive systems.

## Data Availability

Data and data description are available from Dryad [88]. Supplementary material is available online [89].
